# Singing-based interventions for motor and non-motor symptoms in Parkinson’s disease: a systematic review

**DOI:** 10.1007/s00415-026-14072-w

**Published:** 2026-11-03

**Authors:** A. Trinchillo, J. Suh, E. Marcuzzo, C. Vitale, S. Li.ttle, Francesca Morgante, L. Ricciardi

**Affiliations:** 1 Department of Medical, Motor and Wellbeing Sciences, “Parthenope” University of Naples, Naples, Italy; 2 ICS Maugeri Hermitage, Naples, Italy; 3 Department of Neurology, University of California San Francisco, San Francisco, CA 94158 USA; 4 Neurology Unit, Movement Disorders Division, Department of Neurosciences, Biomedicine and Movement Sciences, University of Verona, Verona, Italy; 5 Neuromodulation and Motor Control Section, Department of Psychology and Neuroscience, School of Health and Medical Sciences, University of London, City St George’s, London, UK

**Keywords:** Parkinson’s disease, Singing therapy, Voice and speech disorders, Non-pharmacological rehabilitation

## Abstract

**Background:**

Parkinson’s disease (PD) is characterized by disabling motor and non-motor symptoms. Although pharmacological and rehabilitation interventions improve many motor and non-motor symptoms, persistent impairments commonly remain in domains, including speech, swallowing, gait, and balance, particularly as the disease progresses. For this reason, over the past few years, alternative rehabilitative approaches, such as singing-based interventions, have been used with the aim of addressing symptoms, such as speech impairment, swallowing difficulties, respiratory dysfunction, and mood.

**Objective:**

We aim to review the evidences on the efficacy of singing interventions on motor and non-motor symptoms in patients with PD.

**Methods:**

A comprehensive literature search of PubMed and Google Scholar was conducted for interventional studies published until December 2025. Results were summarized into five symptom domains: motor function, voice quality, breathing, swallowing, and quality of life.

**Results:**

Twenty-six studies involving 1057 patients with PD were included. Singing-based interventions were associated with improvements in motor symptoms, gait, posture, voice quality, respiratory control, and maximum phonation time. Several studies also reported enhanced psychological well-being, social connectedness, and quality of life, with reductions in stress, anxiety, and depressive symptoms. Evidence regarding swallowing outcomes was limited and inconsistent. Considerable heterogeneity in study design, intervention protocols, and outcome measures precluded meta-analysis.

**Conclusion:**

Singing is a promising adjunct therapy for PD rehabilitation, particularly in domains less responsive to pharmacological treatment. Further high-quality research is needed to establish standardized protocols and assess long-term benefits.

**Supplementary Information:**

The online version contains supplementary material available at https://doi.org/10.1007/s00415-026-14072-w.

## Introduction

Parkinson’s disease (PD) is a chronic condition characterized by cardinal motor symptoms, such as rigidity, tremor, and bradykinesia (slowness of movement) [1, 2]. In addition to these well-known motor dysfunctions, non-motor features are present and may precede motor ones. Among the non-motor symptoms, the most common are mood changes, including anxiety, apathy, and depression, as well as autonomic disturbances such as urinary urgency and constipation [3–5]. Moreover, impairment in voice and speech quality and reduction of facial expressivity are common, impacting people’s personal and social quality of life. [6].

Because of the combination of these heterogeneous clinical features, PD should be regarded as a complex, wide-spectrum disease that requires a comprehensive, multidimensional evaluation and management of the patient. [7, 8].

Despite the availability of pharmacological treatments for PD, significant limitations remain in addressing the full spectrum of symptoms, particularly in the domains of non-motor functioning and axial symptoms which include speech, swallowing, gait, and balance issues [8, 9]. Over the last few years, several studies have shown that rehabilitative therapy, including physical exercise, art, dance, and music therapy, has a good impact on those PD manifestations less responding to Levodopa [1].

Over the past two decades, a substantial evidence base has established multidisciplinary rehabilitation as an integral component of PD management. Physical therapy is recommended as a first-line intervention for improving gait, balance, mobility and motor function, with high-quality evidence from systematic reviews, meta-analyses, and the International Parkinson and Movement Disorder Society (MDS) evidence review supporting its efficacy [10]. Occupational therapy also contributes to improvements in activities of daily living, participation, and quality of life, particularly when delivered within multidisciplinary rehabilitation programs [10]. Speech rehabilitation is similarly supported by a robust evidence base. Lee Silverman Voice Treatment (LSVT LOUD), evaluated in five randomized-controlled trials and subsequent systematic reviews and meta-analyses, has demonstrated significant improvements in vocal loudness, speech intelligibility, and communicative participation [11]. Importantly, LSVT LOUD is based on principles of intensive amplitude-oriented motor training that emphasize motor skill acquisition, recalibration of self-perceived vocal effort through sensorimotor integration, and neuroplasticity, rather than strengthening the speech musculature alone. Swallowing rehabilitation has also advanced, with behavioral interventions such as expiratory muscle strength training demonstrating improvements in cough effectiveness, respiratory muscle performance, and selected swallowing outcomes, although the certainty of evidence remains lower than for speech rehabilitation because of the relatively small number of high-quality trials [12]. Despite these advances, many individuals with PD continue to experience persistent communication, swallowing and mobility limitations, incomplete response to rehabilitation, or reduced access to specialist services as the disease progresses. Consequently, complementary interventions such as singing remain of interest. Music engages distributed auditory, motor, cognitive, and limbic networks, and may support rehabilitation through auditory–motor coupling, rhythmic cueing, reward, motor learning, and activity-dependent neuroplasticity [13]. Singing represents a particularly multimodal form of musical activity, integrating respiratory control, phonation, articulation, posture, auditory feedback, cognitive engagement, and social participation, and may therefore complement established evidence-based rehabilitation approaches.

However, there is still fragmented evidence about singing therapy in PD, with studies varying in design, outcome measures, and sample characteristics. The available studies aimed to evaluate the effects of singing on various aspects of PD, including motor function, voice quality, swallowing, and quality of life (QoL). To assess the evidence of the effect of singing on PD symptoms and the main outcome measures used to date in the different studies, we conducted a systematic literature review. Specifically, the present review focuses on the effects of singing on both motor and non-motor symptoms, as well as its overall impact on patients’ QoL. By synthesizing the available evidence, the study seeks to provide a comprehensive understanding of the potential benefits and limitations of singing therapy in PD rehabilitation.

## Methods

We conducted a systematic review of all available studies on singing interventions in patients with PD. A systematic search was performed using two electronic databases—PubMed and Google Scholar. Search covered the period until 30th December 2025, to capture the full development and evolution of singing-based interventions in PD. Relevant keywords and Medical Subject Headings (MeSH) terms, including “Parkinson’s disease,” “sing,” “singing,” “voice,” and “music,” were used for the initial search. We also integrated our research with a manual screening of all references lists kept from retrieved articles, to capture any study not identified during the database search and ensure the inclusion of all potentially relevant studies.

Inclusion and exclusion criteria were defined to guide the selection of studies for this systematic review. Studies were included in this review if they:

1. Explicitly examined the effects of singing interventions in PD;
2. Were published in peer-reviewed journals in the English language;
3. Presented original interventional data (e.g., randomized-controlled trials, cohort studies, pilot studies, case reports, or qualitative research)

We excluded studies that were reviews or meta-analyses, conference abstracts, editorials, commentaries, or letters to the editor.

Methodological characteristics of included studies were evaluated using the Joanna Briggs Institute (JBI) critical appraisal frameworks appropriate to each study design. Given the substantial heterogeneity of study designs and the exploratory nature of many included studies, the appraisal was used to inform interpretation of the evidence rather than to generate quantitative quality scores or exclude studies. A summary of the methodological strengths and limitations of each study is provided in Supplementary Table 1.

For each of the selected study, we collected the following data: methods, sample size, presence of a control group, duration of the intervention, and outcome measures.

We have organized the results into five thematic categories, to perform a structured synthesis of the mentioned articles and facilitate the classification of all measured outcome measures. Indeed, each category corresponded to a specific domain of PD symptomatology targeted by singing-based interventions. We identified the following categories: [1] Singing and Motor Functions, [2] Singing and Voice Quality, [3] Singing and Breathing Functions, [4] Singing and Swallowing, and [5] Singing, sspsychological well-being and Quality of Life (QoL).

## Results

**Fig. 1:**
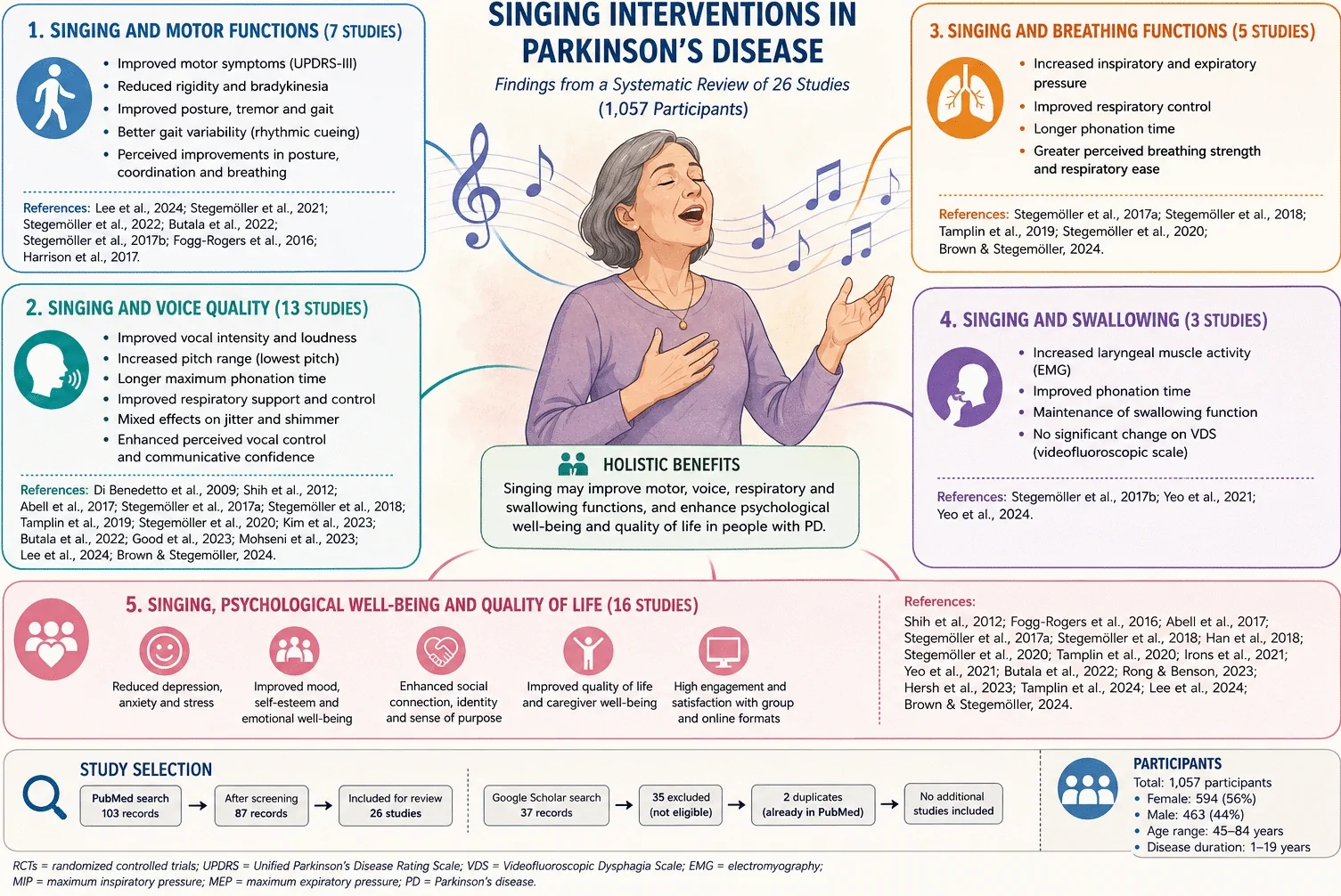
Summary of singing-based interventions in Parkinson’s disease. Overview of the main findings from the 26 studies included in this systematic review, highlighting the effects of singing interventions on motor function, voice quality, breathing, swallowing, and psychological well-being/quality of life in people with Parkinson’s disease. References supporting each category are included within the figure.This figure was created with the assistance of AI-based image generation tools and reviewed by the authors for accuracy

In PubMed, the initial search yielded 103 articles. After removing studies unrelated to singing interventions and screening for eligibility, 87 articles remained. Of these, 26 were selected for review based on the above-outlined inclusion and exclusion criteria. The Google Scholar search returned 37 articles; however, 35 of them did not meet the inclusion criteria. The other 2 articles retrieved from Google Scholar were duplicates already identified in the PubMed search. Hence, no articles from the Google Scholar search were included (Fig. 1).

A total of 26 studies met the inclusion criteria and were analyzed (see Tables 1 and 2 for full details and Fig. 1). A total number of 1057 participants took part in the different studies. Sample sizes in each study were generally small. Participants’ age ranged from 45 to 84, and disease duration from 1 to 19 years. Overall, 594 participants were female and 463 were male.

**Table 1:** Categories of analysis and number of published articles

Category	Number of studies	Authors and years of publication
Singing and motor functions	7	Fogg-Rogers et al. (2016); Harrison et al. (2017); Stegemöller et al. (2017b); Stegemöller et al. (2021); Butala et al. (2022); Stegemöller et al. (2022); Lee et al. (2024)
Singing and voice quality	13	Di Benedetto et al. (2009); Shih et al. (2012); Abell et al. (2017); Stegemöller et al. (2017a); Stegemöller et al. (2018); Tamplin et al. (2019); Stegemöller et al. (2020); Kim et al. (2023); Butala et al. (2022); Good et al. (2023); Mohseni et al. (2023); Lee et al. (2024); Brown and Stegemöller (2024)
Singing and breathing functions	5	Stegemöller et al. (2017a); Stegemöller et al. (2018); Tamplin et al. (2019); Stegemöller et al. (2020); Brown and Stegemöller (2024)
Singing and swallowing	3	Stegemöller et al. (2018); Yeo et al. (2021); Yeo et al. (2024)
Singing, psychological well-being and quality of life	16	Shih et al. (2012); Fogg-Rogers et al. (2016); Abell et al. (2017); Stegemöller et al. (2017a); Stegemöller et al. (2018); Han et al. (2018); Stegemöller et al. (2020); Tamplin et al. (2020); Irons et al. (2021); Yeo et al. (2021); Butala et al. (2022); Rong and Benson (2023); Hersh et al. (2023); Tamplin et al. (2024); Lee et al. (2024); Brown and Stegemöller (2024)

**Table 2:** Details of the 26 studies included in the systematic review

Author/ year	N of participants (m/f)	Mean disease duration	Mean UPDRS III	Study length	Singing protocol	Outcomes evaluated	Significant results (with p values):
Di Benedetto et al., 2009	20 PD patients (13 males and 7 females)	7 years (SD 4.1)	26	Oct 2003 to Feb 2004 20 h of speech therapy and 26 h of choral singing	VCST program included a propaedeutic phase of speech therapy focused on muscular relaxation, respiratory coordination, laryngeal exercises, and prosodic simulation This was followed by choral singing using rhythmic popular and liturgical chants accompanied by a piano, utilizing visual, acoustic (metronome), and proprioceptive cues	UPDRS (Parts I, II, and III) and Hoehn and Yahr scale Otolaryngological: Laryngeal videostroboscopy to evaluate vocal fold vibration and glottic closure Voice and Speech: Acoustic analysis (frequency, jitter, shimmer, etc.) and Maximum Phonation Time (MPT) Auditory Quality: Prosodia and fatigue during reading and monologue evaluated via a Visual Analog Scale (VAS) Respiratory Function: Spirometry (FVC, FEV1), lung volumes (FRC), and maximal static mouth pressures (MIP and MEP)	Functional Residual Capacity (FRC%): Improved (*p* = 0.033) Maximum Inspiratory Pressure (MIP): Improved (*p* = 0.019) Maximum Expiratory Pressure (MEP): Improved (*p* = 0.006) Maximum Phonation Time (MPT): Improved (*p* = 0.000) Prosodia (reading a passage): Improved (*p* = 0.046) Fatigue (reading a passage): Improved (*p* = 0.05)
Shih et al., 2012	13 PD patients (11 males and 2 females)	9 years (SD 9.5)	26.2	Twelve 90-min weekly session	The sessions were led by a voice/speech therapist and singing instructor Each session typically included: 10 min of stretching and gross-motor movement exercises 10 min of breathing training and structured vocal exercises 70 min of singing popular songs	Primary Outcome: Vocal loudness measured by Sound Pressure Level (SPL) at 50 cm during connected speech (reading the "Rainbow Passage") Secondary Outcomes: Pitch range, maximum phonation time, maximum cued volume, average speaking fundamental frequency (F0), and s/z ratio Subject-Rated Measures: Voice Handicap Index (VHI) and Voice-Related Quality of Life (VRQOL)	Primary Outcome (SPL): Not significant (*p* = 0.71) Voice Handicap Index (VHI): Not significant (*p* = 0.84) Voice-Related Quality of Life (VRQOL): Not significant (*p* = 0.90) Correlation: a significant correlation between VHI composite scores and UPDRS I (mental state/non-motor) scores (*r* = 0.771, *p* = 0.04)
Fogg-Rogers et al., 2016	23 participants: 6 PD patients (4 males and 2 females) 8 people with stroke (4 males and 4 females) 9 significant others	Combined group of stroke and PD participants, the mean time since diagnosis was 5.8 years (SD 3.8)	N/A UPDRS Part II (Motor Experiences of Daily Living), where the PD group had a mean score of 21.0	The choir had been running for 2 years, and individual participant attendance ranged from 6 months to 2 years	Specific Choral Singing Therapy (CST) protocol It was a community-based approach led by a Registered Music Therapist and drawing on neuroscience, speech-language therapy, and therapeutic singing principles	Primary: Qualitative experiences and factors influencing participation explored via semi-structured interviews Descriptive Quantitative Measures: Aphasia severity (WAB-R), PD motor experiences (MDS-UPDRS Part II), Health-related Quality of Life (WHOQOL-BREF and WHOQOL-DIS), and social participation (SIPSO)	Quality of Life: The mean WHOQOL-DIS score for the sample was 3.96, which was noted as being higher than published normative data for people with disabilities (3.08), though this was not a comparative statistical test of the intervention’s effect
Abell et al., 2017	11 PD patients (8 males and 3 females)	6.3 years	N/A Hoehn and Yahr (HY) staging scale (participants were Stages I–III) and the Non-Motor Symptom Scale (NMSS) to assess symptom severity	Average of 12 months (with a minimum of 6 months)	The "Shake, Rattle and Roll Choir" was an á cappella group led by a qualified choir maestro Weekly sessions included a 20-min warm-up (deep breathing, stretching, and vocal exercises) followed by 90 min of singing popular repertoire	Primary: Participants’ perceptions of health-related quality of life (HRQoL) captured through semi-structured interviews and analyzed via Interpretive Phenomenological Analysis (IPA) Quantitative Screening/Descriptive Tools: Standardized Mini-Mental State Examination (SMMSE), Hospital Anxiety and Depression Scale (HADS), Hoehn and Yahr scale, Non-Motor Symptom Scale (NMSS), and the Parkinson’s Disease Questionnaire−39 (PDQ-39)	The study identified six categories of positive effects: physical, mood, cognitive functioning, social connectedness, "flow-on" effects, and sense-of-self, with all participants reporting benefits in at least four of these areas
Stegemöller et al., 2016	27 PD patients (10 males and 17 females)	N/A	The low-dosage group had a mean score of 56 (SD 19), and the high-dosage group had a mean score of 58 (SD 17)	8-week singing intervention	Sessions were 60 min long, led by board-certified music therapists. The protocol included: Physical movements for relaxation (trunk flexion, shoulder shrugs, and neck movements) Abdominal breathing exercises to improve projection and breath control Vocal exercises including lip buzzing/trills, glissandos (pitch glides), messa di voce (intensity control), and articulation exercises using specific syllables Group singing of familiar popular songs such as "You are my Sunshine" and "Mairzy Doats"	Voice measures: Phonation duration ("ah" and "ee"), phonation range (pitch glides), and vocal intensity Respiratory measures: Maximum Inspiratory Pressure (MIP) and Maximum Expiratory Pressure (MEP) Quality of Life (QOL): Voice-Related Quality of Life (V-RQOL) and the WHOQOL-BREF (whole health QOL)	Maximum Inspiratory Pressure (MIP): Improved significantly (*p* = 0.049) Maximum Expiratory Pressure (MEP): Improved significantly (*p* = 0.005) Phonation duration (for "ah"): Improved significantly (*p* = 0.05) Voice-Related Quality of Life: Improved significantly (*p* = 0.008) WHOQOL (Whole Health): Improved significantly (*p* < 0.001)
Harrison et al., 2017	23 PD patients (13 males and 10 females)	3.8 years (SD 4.2)	30.5	Single-session study	Participants walked under five different cued conditions, including singing aloud "Row, Row, Row Your Boat" (chosen for its familiarity and beat saliency) at a tempo adjusted to match each individual’s preferred cadence	Gait patterns were measured using an instrumented walkway to assess velocity, cadence, and stride length, as well as the variability of step time, single support time, and step length	Step Time Variability: Singing was significantly less variable than the verbal dual-task (*p* = 0.003) and singing along with music (*p* = 0.008) Single Support Time Variability: Singing was significantly less variable than the verbal dual-task (*p* = 0.004) and singing along with music (*p* = 0.031)
Stegemöller et al., 2017	24 PD patients (8 males and 16 females)	6 years (SD 5.0) for the Low Dosage group and 10 years (with a standard deviation of 6) for the High Dosage group	Motor UPDRS (Part III) score was 27 (SD 11) for the Low Dosage group and 28 (SD 12) for the High Dosage group	Eight weeks	The intervention consisted of group therapeutic singing sessions led by a board-certified music therapist. Each session followed a standardized protocol of warm-up exercises designed to target vocal and respiratory musculature, followed by group singing	Swallowing Physiology: Surface electromyography (EMG) was used to measure muscle activity (peak amplitude, area under the curve, and timing/duration) in the laryngeal and submental muscle groups during thin (water) and thick (pudding) swallows Clinical PD Symptoms: Total UPDRS and Motor UPDRS scores, as well as specific neck rigidity and swallow scores Quality of Life: Swallow Quality of Life (SWAL-QOL) questionnaire	EMG Timing (Laryngeal Muscle Group): Significant increases in Rise Time (Thin: *p* = 0.02; Thick: *p* = 0.01), Fall Time (Thin and Thick: *p* = 0.001), and EMG Duration (Thin: *p* < 0.001; Thick: *p* < 0.001) EMG Timing (Submental Muscle Group): Significant increases in Fall Time (Thin: *p* = 0.04; Thick: *p* = 0.01) and EMG Duration (Thin: *p* < 0.001; Thick: *p* = 0.004) Total UPDRS Score: Improved significantly, reducing by 15% (*p* < 0.001) Motor UPDRS Score: Improved significantly (*p* < 0.001) UPDRS Neck Rigidity Score: Showed a significant increase (*p* = 0.03)
Stegemöller et al., 2017	20 PD patients (7 males and 13 females)	9.15 years	UPDRS III N/A UPDRS Total Scores, range 32–97	4–6 months after participants had completed an 8-week group singing intervention	Vocal Exercises: Lip buzzing (for respiratory control), glissandos (for frequency range), messa di voce (for vocal intensity), and articulation exercises using specific syllables Group Singing: Singing familiar songs like "You Are My Sunshine" (to target phrase length and breath control) and "Mairzy Doats" (for articulation and frequency range) Home Practice: Participants were provided with a practice CD and instructed to exercise at home twice per week	Interviews covering: Program Enrollment: Motivations for joining (e.g., commitment to PD research, improving QOL) Program Engagement: Enthusiasm and social experiences during sessions Program Evaluation: Perceived benefits, non-beneficial aspects, and suggestions for improvement	Participants regarded their involvement in the study as mutually beneficial, fun, and engaging (content analysis procedures)
Han et al., 2018	9 PD patients (3 males and 6 females)	4.8 years (SD 3.0)	UPDRS III N/A UPDRS speech score (item 18)= 1.1 (SD 0.8) and mean Hoehn and Yahr stage 2 (SD 0.5	Six 50-min individual sessions conducted over a period of two weeks. A follow-up test was administered 6 months later	The Individual Therapeutic Singing Program for PD (ITSP-PD) used a progressive approach with four steps per session: a 5-min pre-interview, 20 min of therapeutic vocalization (breathing, phonation, and laryngeal exercises), 20 min of vocal improvisation and song-making, and a 5-min post-interview	Vocal Attributes: Maximum Phonation Time (MPT) via the Praat test Subjective Voice Impact: Voice Handicap Index (VHI) and Voice-Related Quality of Life (V-RQOL) Psychological State: Geriatric Depression Scale (GDS) Qualitative Data: Behavioral observations and interactions	Maximum Phonation Time (MPT): Significantly improved between pre- and post-tests (*p* = 0.011) and remained significant at the 6-month follow-up (*p* = 0.030) Voice Handicap Index (VHI): Significantly lower (improved) in the post-test (*p* = 0.035) Geriatric Depression Scale (GDS): Significantly lower in the post-test (*p* = 0.018) and at the 6-month follow-up (*p* = 0.009) Voice-Related Quality of Life (V-RQOL): Significant improvement found at the 6-month follow-up (*p* = 0.008)
Tamplin et al., 2019	75 PD patients (46 males and 29 females)	8.9 years (SD 6.1)	UPDRS III N/A Mean MDS-UPDRS Total score at baseline as 73.2. It does not provide a single mean score for Part III (Motor) for the entire cohort	3 months	The "ParkinSong" intervention sessions were 2 h long and included 30 min of high-intensity vocal and respiratory exercises 60 min of singing popular and traditional songs 30 min of social interaction and conversation practice	Primary: Vocal intensity (measured via sound pressure level in dB) during a monologue and a loud sentence task Secondary: Speech intelligibility (Speech Intelligibility Test and perceptual ratings), maximum phonation length, maximum respiratory pressures (MIP, MEP, and SNIP), and voice-related quality of life (Voice Activity and Participation Profile—VAPP)	Vocal Intensity (Monologue): Improved significantly (*p* =.018) Vocal Intensity (Loud Sentence): Significant group differences found (*p* =.001) Maximum Expiratory Pressure (MEP): Improved significantly (*p* =.032) Voice-Related Quality of Life (VAPP Total Score): Improved significantly (*p* =.043) Speech Intelligibility (SIT): Significant differences found between groups (*p* =.027) Perceptual Intelligibility: Significant differences found between groups (*p* =.001) VAPP Emotional subscale: Significant difference (*p* =.001) VAPP Social Communication subscale: Significant difference (*p* =.044)
Stegemöller et al., 2019	8 PD patients (7 males and 1 female)	9.3 years	UPDRS III mean 32.8	8 weeks	8 Participants completed a prerecorded group therapeutic singing (GTS) program . Each session included vocal exercises targeting breath support, vocal intensity, and frequency range, followed by the group singing of familiar songs	Voice measures: Phonation duration (sustaining "ah" and "ee"), phonation range (lowest to highest pitch), and vocal intensity Respiratory measures: Maximal Inspiratory Pressure (MIP) and Maximum Expiratory Pressure (MEP)	Maximum Expiratory Pressure (MEP): Improved significantly (*p* = 0.008) Maximum Inspiratory Pressure (MIP): Improved significantly for this pilot study (*p* = 0.06, with significance set at *α* = 0.1)
Tamplin et al., 2020	75 PD patients and 44 caregivers were recruited PD: 46 males and 29 females Caregivers: 14 males and 30 females	9 years (ranging from 1 to 26 years)	UPDRS III N/A Mean MDS-UPDRS Total score at baseline as 73.2 (SD 29.6)	12 months	The "ParkinSong" interdisciplinary protocol consisted of 2-h sessions including: 30 min of vocal warm-ups and high-effort exercises targeting respiratory control, vocal loudness, articulation, and pitch control 60 min of singing activities using familiar songs and rounds 30 min of social interaction and conversation practice	Primary Outcome: Vocal loudness (intensity) during conversational speech (monologue) PD-Specific Secondary Outcomes: Speech intelligibility (Speech Intelligibility Test and perceptual ratings via Direct Magnitude Estimation), maximum phonation time, respiratory muscle strength (MIP and MEP), and voice-related quality of life (Voice Activity and Participation Profile—VAPP) Well-being Outcomes: Depression, Anxiety and Stress Scale (DASS), Quality of Carer Patient Relationship (QCPR) scale, Euroqol-5 Dimensions (EQ-5D), and the Affect Grid	Vocal Loudness (Monologue): Improved significantly overall (*p* = 0.032); at 12 months, weekly singers were 5.13 dB louder (*p* = 0.044) and monthly singers were 5.69 dB louder (*p* = 0.015) than monthly controls Perceptual Speech Intelligibility: Significant group main effect (*p* = 0.001), primarily due to a large decrease in the monthly control group Voice-Related Quality of Life (VAPP Total): Improved significantly for weekly singers (*p* = 0.039) Anxiety (DASS-A): Significant group main effect (*p* = 0.037); weekly singers improved significantly (*p* = 0.010) Affect (Weekly Singers): Significant increases in both valence (*p* < 0.001) and arousal (*p* < 0.001) from pre- to post-session Caregiver Depression (DASS-D): Significant difference at 12 months between weekly singing caregivers and monthly controls (*p* = 0.031) Caregiver Stress (DASS-S): Weekly singing caregivers had significantly lower stress than weekly controls (*p* = 0.044) and monthly controls (*p* = 0.022) at 12 months
Irons et al., 2021	95 PD patients (43 males and 52 females)	7.4 years (Australia), 4.0 years (South Korea), and 8.7 years (UK)	UPDRS III N/A The source does not provide a mean score for UPDRS Part III (Motor); it focuses on patient-reported quality of life and mental health measures	6-month	Singing Protocol Applied: The "Sing to Beat Parkinson’s (STBP)" program consisted of weekly 60-min sessions Each session followed six stages: Welcome and hello song (5 min) Breathing exercises (5 min) Vocal warm-ups (5 min) Warm-up songs such as rounds (10 min) Sharing time (5 min) Preferred song singing (30 min) . The protocol emphasized abdominal breathing, physical movements (e.g., clapping, marching), and practicing speech parameters like voice projection and pitch expansion	Health-Related Quality of Life: Parkinson’s Disease Questionnaire−39 (PDQ-39), covering eight domains including Mobility, Activities of Daily Living (ADL), Emotional Well-being, Stigma, Social Support, Cognition, Communication, and Bodily Discomfort Mental Health: Modified Depression Anxiety Stress Scale (DASS), measuring depression, anxiety, and stress	Stigma (PDQ-39): Improved significantly over time (*p* =.02) Social Support (PDQ-39): Improved significantly over time (*p* =.01), although this was moderated by country and only showed significant improvement in South Korean participants (*p* <.05) Anxiety (DASS): Decreased significantly (*p* =.004) Stress (DASS): Decreased significantly (*p* =.03) Activities of Daily Living (ADL): Significant Time by Country interaction (*p* <.001); ADL worsened for UK participants while remaining stable for South Korean and Australian participants ADL Gender Difference: Significant Time by Gender interaction (*p* =.02); the baseline difference where men had worse ADL than women was absent post-intervention
Stegemöller et al., 2021	25 PD patients (11 males and 14 females)	7.5 years (SD 1.15) for the singing group and 6.25 years (SD 1.54) for the control group	UPDRS III 33.35 (SD 1.71) for the singing group and 26.00 (SD 4.08) for the control group	Single 1-h session	1-h group therapeutic singing (GTS) session followed a specific structure: Greeting song (~ 5 min) Vocal exercises including diaphragmatic breathing, lip buzzing, glissandos, and articulation exercises (~ 15 min) Targeted songs for pitch, articulation, and breath support (~ 15 min) Participant-chosen songs (~ 20 min) Closing song (~ 5 min)	Primary: Cortisol levels and MDS-UPDRS III Secondary: Heart rate, blood pressure, and subjective mood ratings (anxiety, anger, happiness, sadness) Quality of life (PDQ-39) and depression (Beck Depression Inventory)	Heart Rate: Increased significantly in the singing group while decreasing in the control group (*p* = 0.04) Subjective Sadness: Showed a trend toward improvement in the singing group, though it did not reach the threshold for significance (*p* = 0.06) Cortisol and MDS-UPDRS III: no significant changes in cortisol (*p* = 0.25) or motor symptoms (*p* = 0.24) following the acute session Predictors: Baseline PDQ-39 scores were found to be a significant predictor of the change in MDS-UPDRS III scores when both groups were combined (*p* = 0.01)
Yeo et al., 2021	4 patients: 2 PD (A and B), and two head and neck cancer (C and D Males: 1 (Patient B) Females: 3 (Patients A, C, and D)	6.1 years (range 5–7)	UPDRS III N/A	12 weeks, with participants attending 30-min individual sessions twice per week	"Singing-Enhanced Swallowing Protocol" was a modified version of a music-based protocol focused on five areas Respiratory muscle relaxation using keyboard-accompanied upper body movements Vocal folds relaxation through single vowel vocalization, humming, and pitch gliding Laryngeal elevation (primary focus) using sequential vocalization of back and front vowels (/ooh/and/i:/) at jumping pitches to strengthen larynx muscles Modified singing for respiratory control using repeated short phrases in preferred songs Modified singing for articulation using target vowels or syllables in songs	Swallowing Function: Videofluoroscopy Swallowing Study (VFSS) analyzed via the Videofluoroscopic Dysphagia Scale (VDS) Speech-Language Function: Maximum Phonation Time (MPT), laryngeal diadochokinesis (L-DDK), alternating motion rate (AMR), and sequential motion rate (SMR) Quality of Life: Korean version of the SWAL-QOL measure Protocol Feasibility: Repetition rate of various protocol components	Swallowing Function: Improved VDS scores for all four patients, particularly for thick solid barium tasks Quality of Life: Total SWAL-QOL scores increased for all patients, with perceived improvements in swallowing burden and symptom frequency Speech-Language Measures: L-DDK and AMR increased for all patients Etiology Difference: MPT increased for the PD group but decreased for the HNC group PD patients required more repetition for laryngeal elevation and respiratory tasks, whereas HNC patients required more repetition for articulation-focused singing
Stegemöller et al., 2022	18 PD patients (11 female and 7 male)	7.5 (SD 4.7)	UPDRS III 40.22 ± 2.14	Single 1-h session	The 1-h Group Therapeutic Singing (GTS) session consisted of: Greeting song (5 min) Vocal exercises (15 min) Therapist-chosen group singing (15 min) Participant-chosen group singing (20 min) Closing song (5 min) Lyrics or music were not provided to participants to encourage proper posture while singing	Clinical motor symptoms were assessed using the MDS-UPDRS Part III (excluding rigidity as assessments were video-recorded), specifically focusing on four domains: speech and facial expression, bradykinesia, gait and posture, and tremor	Gait and Posture: Improved significantly (*p* = 0.03) Tremor: Improved significantly (*p* = 0.05) Bradykinesia Correlation: There was a significant negative correlation between years singing and the change in bradykinesia score (*r* = − 0.47, *p* = 0.049), indicating that those who had participated in singing for a longer duration showed greater improvement Gait and Posture Correlation: There was a significant positive correlation between years singing and the change in gait and posture score (*r* = 0.56, *p* = 0.016), where those with less singing experience showed greater immediate improvement in this domain Total Motor Score: Showed a trend toward improvement but did not reach statistical significance (*p* = 0.09)
Butala et al., 2022	26 PD patients (16 males and 10 females)	N/A	MDS-UPDRS Part III at baseline 42.2 for the singing-first group (Group S) and 41.5 for the discussion-first group (Group D)	30 weeks, comprising two 12-week intervention periods separated by a crossover point	90-min choral sessions led by a professional choral director The protocol included: 10-min full-body warm-ups (stretching, trunk rotation) Teaching of solfege using Curwen hand signs and the Kodály method Estill voice training principles ("Figures for Voice") to develop specific vocal skills A repertoire of popular and traditional songs such as "Auld Lang Syne" and "Yellow Submarine"	Objective Voice Measures: Volume/loudness (dB), held vowel duration, jitter, shimmer, and harmonic-to-noise ratio (HNR) Clinical and QoL Scales: MDS-UPDRS, Parkinson’s Disease Questionnaire−39 (PDQ-39), Voice-Related Quality of Life (VRQOL), Montreal Cognitive Assessment (MoCA), Geriatric Depression Scale (GDS), Lorig self-efficacy scale, and Short Form-36 (SF-36)	Mean Loudness (Cookie Theft picture description): Significantly improved at 24 weeks (*p* = 0.004) Minimal Loudness (picture description): Improved at 24 weeks (*p* = 0.001) and 30 weeks (*p* = 0.001) MDS-UPDRS Part III (Motor): Significantly improved at 24 weeks (*p* = 0.002) and 30 weeks (*p* = 0.001) in the intention-to-treat analysis of the overall cohort PDQ-39 Emotional Well-Being: Improved significantly from baseline to week 24 (*p* = 0.037) PDQ-39 Body Discomfort: Improved significantly from baseline to week 24 (*p* = 0.001) PDQ-39 Communication: The singing-first group showed significantly greater improvement than the discussion group after 12 weeks of singing (*p* = 0.016) PDQ-39 Stigma: Improved significantly from baseline to week 12 (*p* = 0.046)
Kim et al., 2023	36 participants: 12 PD patients (10 males and 2 females) 12 people with Cerebellar Disease (CD): (9 males and 3 females) 12 Healthy Controls: (6 males and 6 females)	PD group, the mean time since diagnosis was 8.1 years (with a standard deviation of 6.7)	UPDRS III N/A	single-session experimental study	The study evaluated four distinct vocal modes: Spontaneous Speech: Narratives based on topic prompts Spoken-to-a-Model: Reading a written model silently and then speaking it naturally Sung-to-a-Model: Reading a written model and singing it to any melody of the participant’s choice Spontaneous Singing: Singing two or three familiar songs chosen from a list (e.g., "Happy Birthday," "Amazing Grace"	Intelligibility: Percent correct scores from orthographic transcriptions provided by 28 healthy listeners Singing Accuracy: Musically trained listeners rated pitch and rhythm fidelity of familiar songs Singing Competency: Healthy listeners rated competence on a 7-point scale Acoustic Measures: Articulation rate, f 0, coefficient of variation, intensity standard deviation, and smoothed cepstral peak prominence (CPPS)	Intelligibility (PD Group): Participants were significantly more intelligible in the sung-to-a-model mode than in the spontaneous mode (*p* <.0001) and the spoken-to-a-model mode (*p* =.0006) Intelligibility (PD vs. CD): Singing enhanced the vocal output of speakers with PD significantly more than those with CD Pitch Accuracy: PD speakers were rated significantly more accurate in pitch than CD speakers (*p* <.001) Rhythm Accuracy: PD speakers were rated significantly more accurate in rhythm than CD speakers (*p* <.001) Singing Competence: PD speakers were rated significantly higher than CD speakers for both sung-to-a-model (*p* =.018) and familiar songs (*p* <.001) Task Effect: Both PD and CD groups showed significant increases in intelligibility during the spoken-to-a-model mode compared with spontaneous speech (*p* <.0001 for both)
Good et al., 2023	22 PD patients Males: 13 (8 in Group A, 5 in Group B) Females: 9 (4 in Group A, 5 in Group B	N/A	UPDRS III N/A	12 weeks	Weekly community-choir sessions across two sites using an "asset-based" approach focused on inclusion and vocal strengthening Group A (Toronto): Led by a professional choir director with a musical theater background and a piano accompanist Group B (Winnipeg): Led by a music therapist using a guitar for accompaniment	Vocal Range: Lowest and highest achievable pitch Phonation: Duration of sustained vowel phonation Vocal Power: Maximum loudness (intensity) Vocal Quality: Jitter (frequency perturbations) and Shimmer (amplitude perturbations)	Lowest Achievable Pitch (Pitch Min): Improved significantly across both groups (*p* < 0.05) Phonation Duration: Improved significantly across both groups (*p* < 0.05) Shimmer: Reduced significantly (improved) across both groups (*p* < 0.001) Loudness (Intensity): Improved significantly for Group B only (*p* < 0.05) Jitter: Reduced significantly (improved) for Group A only (*p* < 0.01)
Mohseni et al., 2023	33 PD patients (25 males and 8 females)	7.15 years (SD 1.48)	UPDRRS III N/A Hoehn and Yahr scale 1.85 (SD 0.57)	4 weeks, consisting of 12 individual tele-rehabilitation sessions (three sessions per week)	Vocal and singing exercises: Semi-occluded exercises, pitch glide, laryngeal tilt, Cuperto, vowel alternation, staccato, vocal adduction, Messa di Voce, vocal articulation, and vocal agility Chanting and repertoire: Singing of songs and exercises focusing on loudness, breathing, fundamental frequency range, and articulation	Primary Outcome: Voice intensity (measured during vowel sustain/a/, reading, and monologue tasks) Secondary Outcomes: Voice Handicap Index (VHI), maximum frequency range (measured in semitones), jitter, and shimmer	Main Effect of Time: All three groups showed significant improvement across all outcome measures after the intervention (p < 0.001), which was maintained at the 3-month follow-up Group Differences: Significant group effects were found for: Voice Intensity: *p* < 0.001 VHI: *p* < 0.001 Maximum Frequency Range: *p* = 0.014 Shimmer: *p* = 0.001 Combination Therapy Superiority: The combination therapy group (speech + singing) significantly outperformed both the single speech and single singing groups in VHI and Shimmer (*p* = 0.038 and *p* < 0.001) It also showed a larger effect than the singing-only group on voice intensity (*p* < 0.001) and maximum frequency range (*p* = 0.048) Jitter: While jitter improved over time for all groups, there was no significant difference in the degree of improvement between the groups (*p* = 0.162)
Rong & Benson 2023	This was a proof-of-concept single-subject case study featuring one female participant	two years	UPDRS III N/A	9 weekly 1-h rehearsals	intergenerational choral singing protocol involved a mixed-age group of 20–25 members, including college students and seniors The participant rehearsed 21 songs of varying rhythmic complexities and participated in exercises for harmonization and counterpoint to improve musicality	Objective: A semi-automatic multidimensional acoustic analysis covering eight domains: prosody, voice quality, pause pattern, tempo, coordination, rhythm, complexity, and vocal tremor Subjective: Patient-reported outcomes using the Voice Handicap Index (VHI) and the World Health Organization Quality of Life (WHOQOL-BREF) assessment	Short-term (1-week post): Improvements were observed in temporal control (reduced variability), prosody (increased pitch and loudness variations), and voice quality (decreased jitter, shimmer, and noise-to-harmonic ratio) Long-term (8 weeks post): Trends of improvement in tempo, coordination, complexity, and prosody were maintained Subjective: The VHI score decreased from 74 at baseline to 59 at the 8-week follow-up, suggesting a trend toward reduced voice handicap
Hersh et al., 2023	8 participants consisting of 4 couples People with PD: 2 males and 2 females Spouses: 4 total	11.13 years (range 5–16)	UPDRS IIII N/A Hoehn and Yahr scale ranging from Stage 2 to 5	10 weeks	The Singing/Songwriting Group Programme (SSWP) included weekly sessions with the following structure: Physical warm-ups (facial exercises, relaxation) Vocal warm-ups (scales) and exercises (sustained phonation, pitch glides) Singing familiar songs chosen by participants Songwriting: Writing song lyrics and providing feedback Social components like morning tea and participant sharing	Weekly formal and informal interviews Field observations of verbal/non-verbal interactions and engagement Thematic analysis of the couple narratives and experiences	Improved Relationships: The shared joy of music and musical reminiscence strengthened the connection between the person with PD and their spouse Emotional Respite: Spouses reported experiencing emotional relief and support through the group Creativity and Agency: The songwriting component allowed for self-expression and novel creativity Physical and Social Benefits: Participants perceived improvements in voice quality and social confidence
Tamplin et al., 2024	28 PD patients (16 males and 12 females)	7.8 years (SD 6.2)	UPDRS III 17.0 (SD 9.2)	12 weeks	The ParkinSong Online protocol was delivered via Zoom and consisted of weekly 90-min sessions co-facilitated by a music therapist and a speech pathologist Sessions included: Breathing and vocal warm-ups (15 min) Speech exercises (10 min) Intensive high-effort singing focusing on respiratory support and loud vocal projection (35 min) Social communication practice in breakout rooms (30 min)	Feasibility/Acceptability: Recruitment and retention rates, attendance (average 89%), and participant/facilitator satisfaction Speech and Voice: Monologue loudness, loud sentence task, reading loudness, smoothed cepstral peak prominence (CPPs), diadochokinetic rate, Maximum Phonation Time (MPT), and composite scores for intelligibility and naturalness Well-being: Dysarthria Impact Scale, DASS-21 (Depression, Anxiety, Stress), Lille Apathy Rating Scale (LARS-SF), and PDQ-39 Clinical Symptoms: Modified MDS-UPDRS (Total and subscales) and Hoehn & Yahr stage	Loud Sentence Intensity (dB): Significantly decreased from 78.3 to 74.6 dB (*p* =.006) Anxiety (DASS): Significantly increased from 3.6 to 4.5 (*p* =.04) MDS-UPDRS Part II (Activities of Daily Living): Significantly increased (worsened) from 21.7 to 23.7 (*p* =.05)
Yeo et al., 2024	17 PD patients were included in the final analysis Intervention Group: 8 participants (5 males and 3 females) Control Group: 9 participants (3 males and 6 females)	8.86 years (SD 2.04) for the intervention group and 8.89 years (SD 1.76) for the control group	UPDRS III N/A Hoehn and Yahr stages 4 and 5	6 weeks	The "Therapeutic singing-induced swallowing exercise" protocol consisted of individual 30-min sessions held twice per week with a music therapist This was provided in addition to conventional physical and occupational therapy	Swallowing safety and risk of aspiration using the Penetration-Aspiration Scale (PAS) and the NIH-Swallowing Safety Scale (NIH-SSS) Swallowing function via the Videofluoroscopic Dysphagia Scale (VDS) Swallowing-related quality of life using the SWAL-QOL questionnaire Physical disability and daily living activities using the Modified Barthel Index	The intervention group maintained stable scores, while the control group showed a statistically significant decline in functional ability on both the PAS and NIH-SSS PAS (Liquid Food): A significant main effect of time was observed overall (*p* = 0.022), driven primarily by the worsening of the control group’s performance over the 6-week period
Lee et al., 2024	27 PD patients (13 males and 14 females)	10.54 years (SD 6.30)	UPDRS III N/A Hoehn & Yahr stage 2.63 (SD 0.90)	Single-session intervention lasting 30 min	The Therapeutic Group Singing (TGS) protocol was led by a board-certified music therapist and included: Vocal and physical warm-ups (~ 15 min) Group singing of 3–5 familiar preferred songs (~ 15 min) One experimental group (SP + TGS) performed straw phonation (a semi-occluded vocal tract exercise) for the first 15 min in place of traditional warm-ups	Objective Voice Quality: Acoustic Voice Quality Index (AVQI) Psychological State: Visual Analogue Mood Scale (VAMS) measuring anxiety, anger, happiness, and sadness Self-Perceived Voice: Visual Analogue Scale (VAS) ratings for voice quality, loudness, and fatigue	Voice Quality (AVQI): Significant improvement for both the TGS-only and SP + TGS groups compared to the control group (*p* =.023) Self-Reported Mood (VAMS): All groups (including controls) showed significant improvements in anxiety, anger, and sadness after the session Self-Reported Voice Quality: Post-session ratings were significantly higher for both singing intervention groups compared to the control group Self-Reported Loudness: Post-session ratings were significantly higher for the SP + TGS group specifically compared to controls
Brown & Stegemöller, 2024	13 PD patients (7 males and 6 females)	9 years (SD 7) for the singing-first group and 8 years (SD 8) for the EMST-first group	UPDRS III N/A MDS-UPDRS Total score 58 (SD 22) (singing-first group) and 49 (SD 19) (EMST-first group)	4-week interventions, separated by a 1-week washout period . Each intervention was performed 5 days per week for approximately 20 min per day	The Therapeutic Singing (TS) protocol was a respiratory strengthening program designed to be completed at home for 20 min daily	Quantitative: Quality of Life (PDQ-39), depression (Geriatric Depression Scale), and anxiety (Parkinson’s Anxiety Scale) Qualitative: Participants’ perceptions, preferences, and experiences (benefits and burdens) evaluated via open-ended interview questions	Quality of Life (PDQ-39): There was a significant main effect of time (*p* = 0.04), specifically a significant decline (worsening) between the baseline and the final visit (*p* = 0.010)

*ADL* activities of daily living, *AMR* alternating motion rate, *AVQI* acoustic voice quality index, *CD* cerebellar disease, *CPPS/CPPs* cepstral peak prominence Smoothed, *CST* choral singing therapy, *DASS/DASS*−21 depression anxiety stress scale, *DASS*-A dass anxiety subscale, *DASS-D* dass depression subscale, *DASS-S DASS* stress subscale, *EMG* electromyography, *EMST* expiratory muscle strength training, *EQ*-*5D* euroqol-5 dimensions (quality-of-life questionnaire), *F0* fundamental frequency, *FEV1* forced expiratory volume in 1 s, *FRC* functional residual capacity, *FVC* forced vital capacity, *GDS* geriatric depression scale, *GTS* group therapeutic singing, *HADS* hospital anxiety and depression scale, *HC* healthy control, *HNC* head and neck cancer, *HNR* harmonic-to-noise ratio, *HRQoL* health-related quality of life, *H&Y/HY* hoehn and yahr scale, *IPA* interpretative phenomenological analysis, *ITSP-PD* individual therapeutic singing program for parkinson’s disease, *L-DDK* laryngeal diadochokinesis, *LARS*-*SF* lille apathy rating scale – short form, *LSVT* LOUD lee silverman voice treatment loud, *MDS*-*UPDRS* movement disorder society-unified parkinson’s disease rating scale, *MEP* maximum (maximal) expiratory pressure, *MIP* maximum (maximal) inspiratory pressure, *MMSE* mini-mental state examination, *MoCA* montreal cognitive assessment, *MPT* maximum phonation time, *N/A* not available/not applicable, *NIH-SSS*: *NIH* swallowing safety scale, *NMSS* non-motor symptom scale, *PAS* penetration-aspiration scale, *PD* parkinson's disease, *PDQ*−39 parkinson’s disease questionnaire−39, *QCPR* quality of carer-patient relationship scale, *QOL* quality of life, *SD* standard deviation, *SF*−36 short form-36 health survey, *SIPSO* subjective index of physical and social outcome, *SIT* speech intelligibility test, *SMR* sequential motion rate, *SNIP* sniff nasal inspiratory pressure, *SPL* sound pressure level, *SSWP* singing/songwriting group programme: *STBP* sing to beat parkinson’s, *SWAL*-*QOL* swallowing quality of life questionnaire, *TG* therapeutic group singing, *TS* therapeutic singing, *UPDRS* unified parkinson’s disease rating scale (Part I: Non-motor experiences of daily living; Part II: Motor experiences of daily living; Part III: Motor examination), *VAMS* visual analogue mood scale, *VAPP* voice activity and participation profile, *VAS* visual analogue scale, *VCST* voice and choral singing treatment, *VDS* videofluoroscopic dysphagia scale, *VFSS* videofluoroscopic swallowing study, *VHI* voice handicap index, *V-RQOL*/*VRQOL* voice-related quality of life, *WAB-R* western aphasia battery-revised, *WHOQOL/WHOQOL-BREF/WHOQOL-DIS*: world health organization quality of life assessment/brief version/disabilities module

When looking at the study design, there was considerable methodological heterogeneity, including 5 randomized-controlled trials (RCTs), 16 open-label or uncontrolled interventional studies, and 5 case reports or qualitative investigations. The methodologies and experimental protocols varied substantially, leading to considerable heterogeneity in terms of interventions, outcome measures, and follow-up durations. Common methodological strengths included the use of validated outcome measures and detailed descriptions of singing interventions. However, several studies were limited by the absence of control groups, lack of randomization or blinding, short follow-up periods, and single-center recruitment, limiting the generalisability of the findings. A summary of the methodological characteristics, strengths, and limitations of each study is provided in Supplementary Table 1.

Because of this heterogeneity, we decided against a formal meta-analysis and we systematically synthesized results, focusing on the main methodological features, quality assessments, and reported outcomes.

Table 2 shows details of the included studies characteristics.

### Singing and motor functions

Seven studies have demonstrated that singing-based interventions may positively affect motor functions in patients with PD. For instance, Lee et al. [14] found that even a single session of therapeutic singing with semi-occluded vocal tract exercises suggested potential alleviations of motor symptoms as measured by the Unified Parkinson’s Disease Rating Scale, Part III (UPDRS-III). In a similar single-session design, Stegemöller et al. [15] reported significant reductions in rigidity and bradykinesia, along with lowered cortisol levels. Expanding on these findings, Stegemöller et al. [16] tested a remote version of this intervention using prerecorded videos over 8 weeks to investigate the effects of a single one-hour group therapeutic singing session and reported statistically significant improvements in posture, tremor, and gait on the MDS-UPDRS.

Further studies show equally promising results. Butala et al. [17] found significant improvements in motor functions after 24 weeks in a randomized crossover trial of patients participating in weekly group singing. In line with this, Stegemöller et al. [18] observed significant improvements in motor scores, with a decrease in UPDRS-III score after 8 weeks, indicating potential neuromuscular benefits of singing-based interventions. Patients also seem to perceive the benefits of singing; indeed, a study [19] reported perceived improvements in posture, movement coordination, and breathing among participants in choral singing therapy.

Singing has also been proven to help gait. For example, Harrison et al. [20] explored singing as a rhythmic cueing method for gait, revealing reduced gait variability, particularly in step time and single support time, during walking while singing.

Overall, these findings suggest that singing may support motor symptom improvements in patients with PD, particularly in fine motor skills, posture and gait.

### Singing and voice quality

Thirteen studies have investigated the impact of singing-based interventions on the voice quality of patients with PD, which include various factors, namely voice intensity, loudness, pitch, phonation time, jitter and shimmer, and, lastly, perceived voice control, and related confidence.

Studies focusing on singing and voice quality more broadly have yielded mixed results. To examine the short-term benefits, Lee et al., (146) examined the effects of semi-occluded vocal tract (SOVT) exercises in combination with singing. SOVT exercises involve exercises which included partial vocal tract occlusion, such as by phonating through a straw, lip trills, or humming, while vocalizing. SOVT exercises aim to improve vocal efficiency by narrowing the vocal tract to reduce vocal fold stress, promote easier and more balanced vibration, and increase acoustic output with less effort. The study findings revealed significant post-session improvements in the Acoustic Voice Quality Index for both the SOVT + singing and singing-only groups, indicating that even a single session can enhance vocal clarity and efficiency.

Shih et al. [21] provided 12 weekly 90-min singing sessions to 15 PD participants but reported no significant changes in vocal quality during natural speech, suggesting that the kind of intervention associated with higher intensity and duration, and the type of therapeutic method may play critical roles in achieving measurable improvements for patients.

Complementing these findings, Butala et al.’s [17] crossover trial (Parkinsonics) found that singing participants demonstrated gains in factors including conversational loudness, suggesting real-world benefits of vocal engagement. In contrast, however, the above-mentioned study by Shih et al. [21] found no significant changes in vocal loudness. Finally, Mohseni et al. [22] compared singing, speech therapy, and a combined approach across three matched groups. Their findings showed that vocal loudness increased most in the speech therapy and combined approach groups, suggesting that singing should be combined with speech therapy to enhance conversational loudness and voice quality overall.

In addition to voice intensity and conversational loudness, two studies also examine how singing interventions affect the pitch control and range of patients with PD. Good et al. [23] assessed two participant groups (*N*=22), patients with PD and age-matched controls, both of whom took part in a 12-week community-choir program, and found improvement in the lowest pitch. Mohseni et al. [22] provided further evidence that pitch range improved in both the singing and combined intervention groups, reiterating the recommendation that singing should be combined with speech therapy to yield better results for patients.

Kim et al. [24] compared singing and speaking tasks in patients with PD and cerebellar disease, finding that PD participants demonstrated better pitch and rhythm accuracy during singing. However, the above-mentioned study by Shih et al. [21] found no significant changes in pitch range following 12 90-min singing sessions, suggesting inconsistency possibly due to different designs and factors such as duration and intensity of the intervention.

Another factor that was investigated by five studies was maximum phonation time (MPT), a key marker of respiratory and vocal endurance. Di Benedetto et al. [25] combined group speech therapy and choral singing over 13 weeks in a cohort of 20 participants and found significant improvement in MPT, subglottic pressure, and prosody during reading tasks, suggesting that singing can enhance both breath support and expressive speech. Similarly, Stegemöller et al. [26] delivered an 8-week in-person singing program to 27 PD patients and reported significant improvements in MPT and respiratory pressure. Subsequently, Stegemöller et al. [27] study found that in eight participants, both MPT and respiratory control improved after engaging in prerecorded singing lessons. In a larger 12-month randomized trial, Tamplin et al. [28] also identified MPT improvements in groups receiving weekly or monthly singing sessions, with statistically significant improvements just in the singing groups, reinforcing the efficacy of consistent vocal practice. Finally, Good et al. [23] found that community-choir groups (*N*=22) also showed significant increases in MPT, further supporting the benefits of singing-based interventions for the phonatory control of patients with PD.

Four studies (mean number of participants 23, duration range 8–24 weeks) also explored jitter and shimmer outcomes in singing-based interventions, with two reporting no significant changes [17, 26] and two showing improvements [22, 23].

Finally, three studies explored perceived vocal control and communicative confidence in PD patients undergoing singing interventions, emphasizing subjective benefits despite limited objective acoustic changes. Brown and Stegemöller [29] found participants preferred singing over expiratory muscle strength training (EMST) for its perceived vocal benefits. Similarly, two studies [30, 31] reported improved vocal strength, intelligibility, and social confidence through qualitative interviews.

### Singing and breathing functions

Assessment of respiratory function was an outcome measures in five studies. Indeed, breathing, lung strength, and diaphragm mobility are often compromised in PD for several reasons including for example deficit in the posture, restrictive ventilatory dysfunctions [32–34]. In light of this, singing protocols are typically designed with the aim of improving diaphragm use and lungs’ capacity.

Stegemöller et al. [26] found significant gains in maximum inspiratory and expiratory pressure, as well as phonation time, after an 8-week singing program, suggesting that singing strengthens the respiratory musculature. This was supported by another study [28] that investigated the impact of weekly singing sessions on patients with PD, reporting significant improvements in maximum expiratory pressure.

Furthermore, there are two studies that highlight the positive qualitative feedback of the perceived impact of therapeutic singing on respiratory functions [29, 30]. They documented positive participant experience in qualitative interviews, with patients reporting improved breathing strength after partaking in expiratory muscle strength training and group singing.

The immediate benefit of remote singing-based interventions for breathing functions has also been highlighted by Stegemöller et al. [27]. The study assessed the feasibility of the remote delivery of an 8-week singing programme, which showed an immediate improvement in the respiratory abilities of patients. Collectively, these studies underline the potential of singing to enhance the respiratory control and breathing functions of patients with PD.

### Singing and swallowing

Three studies investigated the effects of singing interventions on swallowing function in patients with PD. Stegemöller et al. [18] evaluated the impact of an eight-week group singing program using surface electromyography (EMG). EMG showed increased laryngeal muscle activity, possibly standing for a major airway protection during swallowing, but none of these observed an improvement in self-reported swallowing.

Yeo et al. [35] implemented a 12-week individualized singing protocol and assessed swallowing using the Videofluoroscopic Dysphagia Scale (VDS). In that study there was an improvement of phonation time, but it was not linked to any videofluoroscopy changes. Another study [36] conducted a controlled trial with advanced-stage PD patients, comparing a 6-week singing-based intervention to a home-based oral motor exercise control group. While the singing group maintained their swallowing function and the control group experienced a decline, no significant difference was found on the VDS.

### Singing, psychological well-being and quality of life

Overall, there are 16 research studies consistently demonstrating that singing interventions can significantly enhance quality of life (QoL) and psychological well-being in PD. Of these projects, seven controlled trials provide strong evidence for these effects. For instance, Tamplin et al. [28] highlighted the importance of intervention frequency, built up on weekly sessions, on the improvement of emotional, communicative, and social interaction, and they made it possible with comparisons with a group which underwent less frequent lessons and control conditions. That result was furtherly investigated by the same group in 2020 [37]. In this case, they showed that weekly group singing sessions not only improved voice-related QoL but also reduced stress and depression among both patients and caregivers. Two shorter-term studies [14, 38] observed significant reductions in depressive symptoms (at the Geriatric Depression Scale, Voice Handicap Index, and Beck Depression Inventory-II).

Also Butala et al. [17] reported sustained improvements in emotional well-being and body discomfort in the “Parkinsonics” crossover trial. Additionally, Pacchetti et al. [39] demonstrated that music therapy, including singing, produced superior outcomes in emotional well-being and daily functioning compared to physical therapy. This was in line with another study [26] reporting a subjective improvement in QoL. Furthermore, seven qualitative and mixed-methods studies highlight the emotional depth and social significance of singing in PD. Abell et al. [31] identified key themes, such as restored identity, enhanced mood, and social connectedness, while Hersh et al. [40] reported that songwriting and singing fostered self-worth and stronger interpersonal bonds. Similarly, Fogg-Rogers et al. [19] found that group singing stabilized emotions and encouraged social participation, and Stegemöller et al. [30] described singing as consistently uplifting and emotionally enriching.

Supporting this, Yeo et al. [35] showed that singing-enhanced swallowing therapy reduced anxiety around eating and communication while boosting confidence. Brown and Stegemöller [29] further emphasized that participants preferred singing over other therapeutic methods due to its enjoyment and emotional benefits. Finally, QoL outcomes and their way of changing were also investigated in relationship with the format of the intervention. Tamplin et al. [41] tested the ParkinSong Online telehealth program and, despite no significant QoL score changes, reported high engagement and satisfaction, suggesting emotional and social benefits. Differently, Rong and Benson [42] proposed intergenerational choirs as a means to foster motivation, identity, and connection. On the contrary, Shih et al. [21] found limited QoL improvements in a small in-person trial, likely due to intervention intensity. Irons et al. (2021) [43] confirmed that singing groups reduce stress, anxiety, and stigma internationally, with particularly strong outcomes in South Korea.

Collectively, the 16 findings underline that singing interventions offer holistic benefits, encompassing emotional, social, and physical domains, which support both psychological well-being and broader QoL for individuals living with PD.

## Discussion

Singing-based interventions represent a promising multimodal and complementary rehabilitation approach for people with PD. In addition to the cardinal motor features of PD, including bradykinesia, rigidity, gait disturbance, and postural instability, speech and voice impairment, dysphagia, respiratory dysfunction, and mood symptoms can substantially affect patients’ quality of life. Although evidence-based rehabilitation approaches, including physical and occupational therapy, behavioral speech therapy, swallowing rehabilitation, and respiratory training, can improve several of these domains, symptoms may persist or progress over the course of the disease [44]. Moreover, speech, swallowing, and other axial symptoms often respond incompletely to dopaminergic therapy and may, in some individuals, be adversely affected by advanced treatments, including deep brain stimulation. These persistent and multidimensional rehabilitation needs support continued investigation of complementary non-pharmacological interventions that may be integrated within multidisciplinary PD care.

Within this context, our findings suggest that singing-based interventions may provide measurable benefits across several domains relevant to PD. Singing simultaneously engages respiratory control, phonation, articulation, auditory feedback, sensorimotor integration, and cognitive and emotional processes, offering a distinctive multimodal form of skilled motor practice. The potential role of singing should therefore not be considered as an alternative to established evidence-based rehabilitation, but rather as a complementary approach that may target overlapping functional domains and provide additional opportunities for intensive, engaging, and socially meaningful practice. Importantly, access to and engagement with singing opportunities may also be shaped by structural and societal factors, as many community-based choirs and singing groups operate outside formal healthcare systems and may not be routinely integrated into or supported by PD care pathways.

The reviewed studies generally showed and highlighted improvements in voice quality (e.g., loudness, pitch range, phonation time), respiratory control and overall QoL [27, 28, 40].

These findings emphasize the utility of singing training in improving not only voice loudness, as initially assumed, but also breathing functions. Such utility, moreover, has been shown to increase when alternative singing rehabilitation is combined with targeted speech, breathing and swallowing-oriented forms of therapy. Doing this, with a customized approach based on patients’ symptoms, would enhance therapeutic outcomes holistically.

Nevertheless, singing training has not yet been established as an official form of therapeutic intervention in PD, due to the lack of consistent results regarding which outcomes benefit most from it and which protocol is best suited for PD. For instance, voice can improve in loudness and intensity, but effects on speech remain unclear. Some improvements have been found in intelligibility and prosody while reading aloud, but no changes during spontaneous or natural speech have been demonstrated [21, 22], raising the question of whether singing has more effects on automatic verbal behaviors. Therefore, more comparative studies are needed, involving PD patients and healthy controls and with a stronger focus on comparing reading with natural speaking, in order to distinguish whether both spontaneous and planned speech would benefit from singing training.

Another gap is the lack of studies comparing speech impairments between different neurodegenerative diseases, for example parkinsonian and non-parkinsonian (e.g., cerebellar or upper motor neuron dysarthrias). Such studies would help understanding possible pathogenetic mechanisms of dysarthria. At the same time, evidence on the impact of singing on swallowing functions remains still unclear since the lack of standardized measures to assess it. Only a few studies have addressed this domain directly [17, 18]; however, they did so without a validated protocol. This gap highlights the need to validate objective methods and outcome measures to assess swallowing and then compare any change before and after a singing training protocol.

Finally, comparisons between online and face-to-face singing interventions should be interpreted with caution, as differences in social interaction, group dynamics, acoustic quality, technological factors, participant engagement, and accessibility may all influence therapeutic outcomes independently of the singing intervention itself.

### Limitations

We specifically aimed to synthesize the evidence on singing-based interventions in PD and was not designed to assess their comparative effectiveness against established rehabilitation approaches. Accordingly, direct comparisons with physical, occupational, speech, swallowing, or respiratory rehabilitation interventions were beyond the scope of the present review. Although the findings should therefore not be interpreted as evidence that singing is equivalent or superior to established evidence-based therapies, they highlight its potential as a complementary, multimodal intervention that may engage overlapping respiratory, phonatory, sensorimotor, cognitive, and psychosocial processes. Future comparative effectiveness and adequately powered controlled studies are needed to determine whether singing provides additional or domain-specific benefits when integrated with established rehabilitation programs.

Another major limitation of the current literature lies in the small sample sizes, most studies involved fewer than 50 participants, and the predominance of non-randomized, non-blinded interventional designs. These study designs not only limit causal inference but also weaken the overall strength of the collected evidence. A formal item-level methodological quality appraisal was not undertaken because of the substantial heterogeneity of the included study designs. Instead, the methodological strengths and limitations of each study were systematically reviewed and summarized (Supplementary Table 1). Consequently, the findings should be interpreted with caution, particularly those derived from small proof-of-concept studies and uncontrolled interventions. These methodological limitations reduce the certainty of the available evidence despite generally encouraging findings.

### Future directions

Therefore, further intervention-development and pilot studies are needed to address current gaps, better define the therapeutic targets, content, and dosage of singing-based interventions, and identify appropriate standardized outcome measures. These foundational data should subsequently inform the design of adequately powered randomized-controlled trials to validate efficacy and minimize bias.

Also, trials comparing PD with other neurological conditions are needed, with the aim of determining disease-specific versus broader neurological mechanisms.

Future research should also investigate the impact of singing-based interventions on caregiver well-being and the patient–caregiver relationship, as these may contribute to the overall therapeutic benefits of singing.

Finally, innovative delivery methods, including tele-rehabilitation and hybrid models, should be explored more to improve accessibility and promote long-term adherence to singing-based interventions.

## Conclusion

In this systematic review, we aimed to point out evidence collected about the use of singing-based interventions as a complementary rehabilitation strategy for patients with PD. Singing can provide benefits in voice quality, respiratory control, and quality of life PD patients, domains often insufficiently addressed by pharmacological therapies. Effects on spontaneous speech intelligibility, prosody, and swallowing remain still unclear, highlighting the need for further refined investigations. Future studies will also need standardized, objective assessment tools and outcome measures. Finally, we can conclude that singing represents a promising, enjoyable and socially engaging intervention that can enrich PD holistic care and address unmet needs in its classical treatment methods.

## Supplementary Information

Below is the link to the electronic supplementary material.

Supplementary file1 (DOCX 34 KB)

Supplementary file2 (DOCX 49 KB)

## Data Availability

All data generated or analyzed during this study are included in this published article.
