# Supplementary material for: Singing-based interventions for motor and non-motor symptoms in Parkinson’s disease: a systematic review
Source: J Neurol. 2026 Nov 3;273(11):697. doi: 10.1007/s00415-026-14072-w (PMC13731696; doi:10.1007/s00415-026-14072-w)
Supplement: Supplementary file 1 [file 415_2026_14072_MOESM1_ESM.docx]

Supplementary Table 1: Study-design classification and narrative methodological appraisal

| **Study** | **Primary design** | **JBI checklist** | **Methodological strengths supported by the report** | **Principal methodological limitations** |
| --- | --- | --- | --- | --- |
| Di Benedetto et al., 2009 | Uncontrolled pre-post pilot | Quasi-experimental | Consecutive referrals; no dropouts; objective respiratory, acoustic and clinical assessments | No comparator or randomisation; one pre/post measurement; no assessor blinding reported |
| Shih et al., 2012 | Single-arm pilot intervention | Quasi-experimental | Defined protocol; objective and patient-reported voice outcomes | Small sample; no comparator; limited information on co-interventions, assessor blinding and attrition handling |
| Fogg-Rogers et al., 2016 | Qualitative interview study | Qualitative research | Semi-structured interviews; participant quotations; ethics and analytic approach reported | Mixed stroke/PD sample; participants already attending choir; researcher positioning/reflexivity incompletely reported |
| Abell et al., 2017 | Interpretive phenomenological study | Qualitative research | IPA congruent with the research question; clear recruitment, interview procedure and participant accounts | Small self-selected established choir sample; researcher cultural/theoretical position and influence not explicitly addressed |
| Stegemöller et al., 2017a | Non-randomised dose-comparison pre-post study | Quasi-experimental | Standardised intervention; low- and high-dose comparison; objective voice/respiratory measures | No non-singing control; non-random dose allocation; single pre/post measurement; small groups |
| Harrison et al., 2017 | Within-participant acute experimental study | Quasi-experimental | Standardised within-person cue conditions; instrumented gait assessment | Single-session laboratory study; no untreated longitudinal control; order/carry-over and assessor masking not fully reported |
| Stegemöller et al., 2017b | Non-randomised dose-comparison pre-post study | Quasi-experimental | Objective swallowing EMG and clinical outcomes; common protocol across dose groups | No non-singing control; non-random allocation; single pre/post measurement; multiple outcomes |
| Stegemöller et al., 2018 | Post-intervention qualitative study | Qualitative research | Qualitative enquiry addressed motivation, engagement and perceived effects; participant perspectives reported | Retrospective accounts from prior intervention participants; researcher reflexivity and analytic detail limited |
| Han et al., 2018 | Single-arm feasibility intervention | Quasi-experimental | Clearly described individual intervention; objective and patient-reported outcomes; six-month assessment | Very small sample; no comparator; incomplete/unclear follow-up; single baseline and post-treatment assessment |
| Tamplin et al., 2019 | Four-arm non-randomised controlled trial | Quasi-experimental | Active controls; blinded speech raters; baseline-adjusted analyses; objective outcomes | Participants selected groups rather than random allocation; potential baseline confounding; one main post-intervention time point |
| Stegemöller et al., 2020 | Single-arm telerehabilitation feasibility study | Quasi-experimental | Standardised prerecorded programme; feasibility and physiological outcomes reported | Very small completer sample; no comparator; attrition; single pre/post assessment; pilot alpha set at 0.10 |
| Tamplin et al., 2020 | Twelve-month non-randomised controlled trial | Quasi-experimental | Active controls; repeated follow-ups; blinded intelligibility ratings; mixed-effects analyses | Self-selection/non-random allocation; assessors not blinded for all outcomes; unequal exposure and residual confounding possible |
| Irons et al., 2021 | International single-arm pre-post study | Quasi-experimental | Large multicountry cohort; standardised programme; validated QoL and mental-health measures | No control group; country/site heterogeneity and self-selection; single pre/post evaluation |
| Stegemöller et al., 2021 | Non-randomised controlled acute study | Quasi-experimental | Quiet-reading control; motor videos rated by two assessors masked to time and condition | Small non-random groups selected by prior singing participation; single acute session; baseline comparability concerns |
| Yeo et al., 2021 | Four-patient case series | Case series | Detailed protocol; objective VFSS and speech-language outcomes; follow-up described | Only four heterogeneous cases, including two with PD; non-consecutive purposive sample; no comparator |
| Stegemöller et al., 2022 | Single-arm acute pre-post study | Quasi-experimental | Standardised session and video-derived motor-domain outcomes | No control group; small sample; single session; one pre/post measurement and multiple comparisons |
| Butala et al., 2022 | Randomised blinded crossover trial | Randomised controlled trial | Random allocation; active comparator; repeated assessments; blinded outcome assessment and intention-to-treat analysis | Small sample; participant/provider blinding impossible; allocation concealment unclear; possible period/carry-over effects |
| Kim et al., 2023 | Comparative cross-sectional experiment | Analytical cross-sectional | Clearly described diagnostic groups and standardised speech/singing tasks; listener-based and acoustic outcomes | Small groups; limited identification/control of confounders; cross-sectional task comparison cannot establish treatment efficacy |
| Good et al., 2023 | Two-site single-arm pre-post study | Quasi-experimental | Objective acoustic outcomes and implementation across two community sites | No non-singing comparator; sites used different facilitators/accompaniment; small sample and multiple testing |
| Mohseni et al., 2023 | Three-arm single-masked RCT | Randomised controlled trial | Random assignment with concealed allocation; masked assessor; no dropout; three-month follow-up | Small groups; participants and therapists unblinded; single-centre study |
| Rong and Benson, 2023 | Single-participant proof-of-concept case report | Case report | Detailed theoretical framework, intervention, acoustic measurement system and two follow-ups | Single participant; adverse events not explicitly reported; no comparator and no causal inference |
| Hersh et al., 2023 | Longitudinal focused ethnography | Qualitative research | Repeated observations and interviews over 10 weeks; couples’ perspectives and direct quotations | Four self-selected couples; researcher position and influence only partly addressed; setting-specific findings |
| Tamplin et al., 2024 | Single-arm online feasibility study | Quasi-experimental | Prospective feasibility outcomes; high attendance/retention; blinded perceptual speech ratings | No control group; efficacy underpowered; single pre/post comparison; some outcomes worsened |
| Yeo et al., 2024 | Prospective intervention with historical matched controls | Quasi-experimental | Matched usual-care controls; identical conventional rehabilitation; objective VFSS outcomes | Non-random allocation and historical controls; small groups; assessor blinding unclear; one pre/post assessment |
| Lee et al., 2024 | Three-arm randomised single-session trial | Randomised controlled trial | True experimental random assignment; speaking-only control; standardised interventions; objective AVQI and mood outcomes | Small unequal groups; allocation concealment and assessor blinding not reported; single-session outcomes only |
| Brown and Stegemöller, 2024 | Randomised crossover mixed-methods trial | Randomized controlled trial and qualitative component | Random intervention order; active comparator; direct within-person comparison; quantitative and qualitative outcomes | Very small sample; participant/provider blinding impossible; concealment/assessor blinding unclear; short washout and possible carry-over; limited qualitative reflexivity |
