# Supplementary material for: Singing-based interventions for motor and non-motor symptoms in Parkinson’s disease: a systematic review
Source: J Neurol. 2026 Nov 3;273(11):697. doi: 10.1007/s00415-026-14072-w (PMC13731696; doi:10.1007/s00415-026-14072-w)
Supplement: Supplementary file 2 [file 415_2026_14072_MOESM2_ESM.docx]

**Identification of studies via databases and registers**

Records removed *before screening*:

Duplicate records removed (n = 2)

Records marked as ineligible by automation tools (N/A)

Records removed for other reasons (N/A )

Records identified from

Databases

1. Pubmed 103
2. Google Scholar 37

**Identification**

Records screened

(n = 140)

Records excluded

(n = 53)

Reports sought for retrieval

(n = 87)

Reports not retrieved

(n = 51)

**Screening**

Reports assessed for eligibility

(n = 36)

Reports excluded: 10

Reason 1 – not singing based interventions (n = 3)

Reason 2 – wrong study design (n = 3)

Reason 3 – outcome not relevant/insufficient (n = 4)

etc.

Studies included in review

(n = 26)

**Included**
